# The bumps and bruises from turning a blind eye: Learning from our failures and surprises

**DOI:** 10.1007/s40037-018-0421-1

**Published:** 2018-04-19

**Authors:** Lara Varpio, Alisa Nagler

**Affiliations:** 1 Department of Medicine, F. Edward Hébert School of Medicine and Uniformed Services University of the Health Sciences, Bethesda, MD USA; 2 American College of Surgeons, Chicago, IL USA; 3 School of Medicine, Duke University, Durham, NC USA

Lord Horatio Nelson, a Vice-Admiral in the British Royal Navy who served during the late 18th century, is celebrated as an inspirational leader [1] and an unparalleled strategist [2]. Historians credit his unusual tactics for securing pivotal naval victories during the Napoleonic Wars [2]. Lord Nelson regularly positioned himself in the throes of battle, and carried the wounds of war as a result (i.e., having lost an eye and most of an arm in combat). While his military career is what secured his status as a British hero, one of the Admiral’s most lasting legacies may be lost to the annals of history since it is not the result of a bold tactic, but of an act of insubordination.

The story goes that, during the 1801 Battle of Copenhagen, Lord Nelson’s commanding officer, Sir Hyde Parker, favoured a cautious battle plan. Lord Nelson did not support a conservative approach. In the heat of battle, a well-aimed cannon ball reduced the mast of Parker’s ship to splinters. To communicate with Nelson’s ship, Parker hoisted signal flags ordering Nelson to disengage from the fight. But Lord Nelson had no intention of breaking off the attack. Lifting his telescope to the eye he lost in battle so long ago and looking to Parker’s ship, Nelson informed his crew that he *truly did not see *any signals. With this act of insubordination, Lord Nelson gave us the idiom: turning a blind eye.

## The damage inflicted when academics turn a blind eye

For Lord Nelson, turning a blind eye to Parker’s orders had beneficial outcomes [4]. The British won the Battle of Copenhagen. Parker was disgracefully recalled to Britain, while Lord Nelson was promoted to Commander-in-Chief of the British fleet.

But turning a blind eye is not always accompanied with benefits. When academic researchers and innovators ignore undesirable information and turn a blind eye to failures and unexpected outcomes, the results are generally detrimental.

Knowledge is rarely developed with ease. Much more often, researchers and innovators toil arduously to devise new experiments, to test hypotheses, to develop new interventions, and to consider new interpretations of complex data. Again and again, scholars face failure and surprises: experiments can generate completely unexpected results, well thought out projects can have unintended consequences, hypotheses can simply be wrong, interventions can have no effect, and meaningful interpretations of data can elude us. All too often, scientific discovery is a game of patience—of relentlessly trying *yet another* experiment, after *yet another* failure. Knowledge is rarely developed at a steady marching pace. Instead, knowledge advances in lurches—in the unsteady, unpredictable movements of scholars who, according to psychologist Daniel Wegner, bumble along, only once in a while discovering something noteworthy [3].

This is why exclaiming *‘Eureka’* is such a passionate cry. Rarely do researchers or innovators get to say it!

Problematically, researchers and innovators have learned to hide their failures and surprises. Modern academic journals generally reserve the pages of their publications for eureka moments, and not for stories of failure or surprise. On the whole, this means that we tend to overvalue success, underestimate the frequency of failures, and hide unexpected findings until we can (if ever) make sense of them. By turning a blind eye to our failures and surprises, we have created a skewed understanding of what it takes to build knowledge and of the value of learning from our non-eureka moments.

Academia has a history of turning a blind eye to these moments—moments that form the majority of a scientist’s career. Johannes Haushofer, a professor at Princeton University, published a CV of Failures in an effort to ‘provide some perspective’ to how many failures lie behind a scientist’s successes [4]. In this CV, Haushofer lists the degree programs that did not accept him, the research funding he was not awarded, the papers that were rejected, and other ‘failures’ that served as stepping-stones for his successful academic career. Reading this alternative CV demonstrates the sheer volume of failures academics face, and why learning from our failures is an important practice to embrace.

But laying bare our faults is not an easy thing to do. Failures and surprises often hurt. Turning a blind eye to them can be an easier and simpler solution. But, if you or anyone you know has ever suffered from complete vision loss in one eye (be it from battle or an unexpected retinal detachment), you know that a blind eye changes everything. When you go from binocular to monocular vision, your depth perception and visual cues shift unexpectedly. Instead of gracefully sitting in the chair, you land with a thud on the floor. Navigating a doorframe results in bruised shoulders and elbows. Such are the visible bumps and bruises of having one blind eye. Similarly, there are negative consequences that result from turning a blind eye to academic failures and surprises. We can become biased towards statistically significant results. The size of our N can become the focus of attention, instead of a meaningful story discovered via few (or even just one) participants. Our colleagues are forced to learn the hard way that which we have already discovered through failure.

Furthermore, revealing failures and surprises publically is not a simple thing to do; admitting failure or unexpected results can have consequences. Will the funding agencies look down on me if I publish my errors? Will my institution deny me promotion if I admit that my teaching innovation was not met with cheers from the students? What will my colleagues think if I reveal how our collaboration ended in failure?

Our time in the health professions education (HPE) community suggests that HPE is ready to support those who lay bare their bumps and bruises. Our experience is that HPE scholars are willing to eschew the blind eye practice, and are willing to learn from errors and surprises.

With this supplement, we put on display some of medical education’s bumps and bruises. Enclosed are stories of failed innovations, unintended consequences, and surprising outcomes. Some of these stories will make you laugh. Others will have you wincing in sympathetic pain. Most will have you nodding along with a ‘been there, done that’ wry smile.

*Gagliardi *and* Rudd* reveal how their determination to complete a study and their willingness to compromise to achieve that goal resulted in a dataset that offered no new insights. *Worden *and* Ait-Daoud Tiouririne* remind us that, even with the most innovative and thoughtful instructional design, the broader societal context influences learner receptiveness to educational efforts, especially when dealing with sensitive topics such as culture and race. *Case, Lewis *and* Pippitt* describe how they took on the challenge of addressing learners’ experiences of shame, and how difficult it was to discuss shame without inducing it. *Czepiel*, a medical student, shares her struggle both to develop and then to dissolve relationships with patients in ways that honour the experiences she shared with them. *Johnson *and* Reid* explain how exploring inequalities and stigma associated with women’s healthcare was met with powerful opposition from learners. *Young* reminds us how important our language is in describing our health professions education research, noting that specificity remains important for shaping and reporting of the research findings, while generalism is vital for translation and application of findings within educational contexts. *Norman* unabashedly details the lessons he has learned from both successes and failures across his career in medical education. *Wilkinson* recounts his evolving understanding of the concept of ‘objectivity’ and urges us to trust the wisdom of experienced raters (Note: he also warns of the pitfalls of voicing a complaint). *Cleland* recounts how she learned to accept that, upon occasion, one must simply abandon original research plans and how such flexibility can allow for new, and perhaps more powerful insights. Finally, *Varpio *and* McCarthy* share the story of a research project that inadvertently revealed the physical and emotional trauma some learners were experiencing during global health electives, causing them to halt a study and bring institutional awareness to the situation.

In developing this supplement, we recognized that these stories would not fit within the standard Introduction/Methods/Results/Discussion (IMRaD) structure of research papers. Instead, we developed a narrative format that would allow authors to share the plot, characters, and consequences of their stories. We are grateful to the dedicated and creative reviewers (see list of Reviewers for this supplement) who so easily adapted to this non-traditional manuscript format and provided rich and meaningful feedback. A special thanks is owed to Dr Meghan Hamwey for collating the reviews, for doing additional reviews when we had very disparate reviewer comments, and for acting as a supporting editor. Finally, we extend our deepest thanks to the authors who were willing to share the stories of their failures and surprises. We will all learn from their bumps and bruises.

Lastly, with an overwhelming volume of submissions (+50), we were disappointed not to be able to include more stories in this supplement. So, in response to what appears to be a welcomed concept, Perspectives on Medical Education is excited to announce Surprise Outcomes/Failed Research as a new Manuscript Category that will welcome submissions for future regular journal editions!

### Reviewers for this supplement

- Rebecca Blanchard
- Peter Boendermaker
- William Bynum
- Ron Cervero
- Jen Cleland
- Tracy Collett
- Janet Corral
- Erik Driessen
- Luba Dumenco
- Timothy Dornan
- Kinga Eliasz
- Rachel Ellaway
- Deborah Engle
- Kevin Eva
- Kristen Goodell
- Laurence Grierson
- Pippa Hall
- Lisa Howley
- Debbie Jaarsma
- Adina Kalet
- Mahan Kulasegaram
- Meghan Loeser
- Jimmie Leppink
- Lorelei Lingard
- Lauren Maggio
- Bonnie Miller
- Robin Ovitsh
- Patricia O’Sullivan
- Johanna Schonrock-Adema
- Renee Stalmeijer
- Sebastian Uijtdehaage
- Cees van der Vleuten
- Shari Whicker
- Tasha Wyatt
- Kal Winston
- Tim Wood
